# Serum exosomal long noncoding RNAs lnc-FAM72D-3 and lnc-EPC1-4 as diagnostic biomarkers for hepatocellular carcinoma

**DOI:** 10.18632/aging.103355

**Published:** 2020-06-18

**Authors:** Zhicheng Yao, Changchang Jia, Yan Tai, Hao Liang, Zhaozhong Zhong, Zhiyong Xiong, Meihai Deng, Qi Zhang

**Affiliations:** 1 Department of General Surgery, The Third Affiliated Hospital of Sun Yat-sen University, Guangzhou 510000, China; 2 Department of Cell-gene Therapy Translational Medicine Research Center, The Third Affiliated Hospital of Sun Yat-sen University, Guangzhou 510000, China; 3 Department of Liver Disease Lab, The Third Affiliated Hospital of Sun Yat-sen University, Guangzhou 510000, China; 4 Department of Hepatobilliary Surgery, The Third Affiliated Hospital of Sun Yat-sen University, Guangzhou 510000, China

**Keywords:** hepatocellular carcinoma, exosomes, lncRNAs, lnc-FAM72D-3, lnc-EPC1-4

## Abstract

Long noncoding RNAs (lncRNAs), such as LINC00462, HOTAIR, and MALAT1, are significantly upregulated in hepatocellular carcinoma (HCC) tissues. However, lncRNA expression in the serum of HCC patients is still unclear. To identify candidate lncRNAs for HCC diagnosis, we purified exosomal total RNA from the serum of healthy volunteers (controls) and hepatitis, cirrhosis, and HCC patients. To assess the function of lncRNAs, small interfering RNAs and overexpression vectors were designed and cell viability and cell apoptosis assays conducted. The exosomes of the control group had a larger number of lncRNAs with a high amount of alternative splicing compared to hepatic disease patients. qPCR assays showed that lnc-FAM72D-3, lnc-GPR89B-15, lncZEB2-19, and lnc-EPC1-4 are differentially expressed in HCC. Furthermore, the expression level of lnc-EPC1-4 correlated with age. While the expression levels of lnc-GPR89B-15 and lnc-EPC1-4 correlated with serum alpha-fetoprotein level. *lnc-FAM72D-3* knockdown decreased cell viability and promoted cell apoptosis, indicating that *lnc-FAM72D-3* functions as an oncogene in HCC. In contrast, *lnc-EPC1-4* overexpression inhibited cell proliferation and induced cell apoptosis, indicating that it functions as a tumor suppressor gene. Collectively, these findings show that lnc-FAM72D-3 and lnc-EPC1-4 play a novel role that might contribute to hepatocarcinogenesis and identify potential candidate biomarkers for HCC diagnosis.

## INTRODUCTION

Liver cancer, also called hepatocellular carcinoma (HCC), is the fifth-most common cancer worldwide, with a mortality rate that increased by almost 3% per year from 2010 to 2014 [1, 2]. Surgical treatment of HCC might be effective in the initial stage of the disease. However, most HCC patients present with intra- and extrahepatic metastasis upon diagnosis, which limits their treatment options to radiotherapy and chemotherapy. Unfortunately, the treatment efficacy of radiotherapy and chemotherapy is limited [3]. Therefore, early diagnosis of HCC is of utmost importance. Currently, the most commonly used methods of diagnosing HCC involve imaging or detection of tumor biomarkers, such as alpha-fetoprotein (AFP), from the patient’s serum. However, these techniques are less sensitive for early diagnosis of HCC [4]. Therefore, it is important to identify novel diagnostic and prognostic molecular markers to improve early diagnosis of HCC.

Long noncoding RNAs (lncRNAs) are non-protein-coding transcripts with a length of >200 nucleotides. lncRNAs participate in various biological processes, such as tumorigenesis, metastasis, and proliferation [5–8], so they are considered diagnostic biomarkers for multiple cancers, such as gastric, bladder, colorectum, prostate, and renal cancers [9–12]. lncRNAs, such as LINC00462, HOTAIR, MALAT1, CCAT1, CCAT2, LINC00161, and SPRY4-IT1, are significantly upregulated in HCC tissues [13–19]. However, lncRNA expression in the serum of HCC patients is still unclear.

Circulating exosomes are small 30–150 nm membrane vesicles that are released into extracellular environments, such as serum or plasma [20]. They usually contain proteins and RNAs that perform various functions, such as cell–cell communication and signaling. Researchers have focused on identifying biomarkers in blood exosomes as a minimally invasive sample source that reflects the signatures of pathological conditions such as cancer and atherosclerosis without the need for surgical sample collection [21]. Although various microRNAs (miRNAs) and proteins are found inside exosomes, indicating that they could potentially be used as diagnostic biomarkers for HCC, little is known about the serum exosomal lncRNAs in HCC [22–24].

This study determined whether exosomal lncRNAs have functions in hepatic diseases and could potentially be used as diagnostic biomarkers. We purified exosomal lncRNAs from 45 healthy volunteers, 45 hepatitis patients, 45 cirrhosis patients, and 45 HCC patients. We conducted RNA sequencing (RNA-seq) to obtain expression profiles and performed correlative and functional analyses of candidate lncRNAs.

## RESULTS

### Exosome particle and protein concentrations modified in hepatic diseases

To investigate exosomal lncRNAs involved in hepatic diseases, we collected serum from healthy volunteers and hepatitis, cirrhosis, and HCC patients. Exosomes were purified by ultracentrifugation, and their sizes were determined by NTA. As shown in Supplementary Figure 1A, particle diameters of exosomes ranged from ~50 to 150 nm for the four groups, and there was no significant difference in the size distribution. CD63, TSG101, and CD9, which are surface antigens commonly expressed in exosomes, were detected in all four groups (Supplementary Figure 1B). In contrast, calnexin protein expression was not detected in any group (Supplementary Figure 1B). These findings indicated successful purification of exosomes and also that they were of uniform size across the four groups.

To further evaluate potential differences between exosomes of healthy volunteers and hepatic disease patients, we measured the particle and protein concentrations of exosomes. The control and hepatitis groups had similar particle concentrations, but the particle concentration was 50% lower for the cirrhosis group and 20% higher for the HCC group (Table 1). The hepatitis, cirrhosis, and HCC groups also had higher protein concentrations compared to the control group, indicating that hepatic diseases induce changes in both particle concentration and protein concentration of exosomes.

**Table 1:** Comparison of serum exosome particle concentrations and protein concentrations in healthy volunteers and patients with hepatic diseases.

**Exosome grouping**	**Particle concentration (particles/ml)**	**Exosome protein concentration (μg/μl)**
**Normal**	3.40E+11	9.127
**Hepatitis**	3.81E+11	11.345 *
**Cirrhosis**	1.97E+11 *	11.399 *
**Hepatocellular carcinoma**	4.54E+11 *	11.299 *

Compared with the control group, * p < 0.05 was considered to be significantly different.

### Bioinformatics study of exosomal lncRNAs

**Figure 1:**
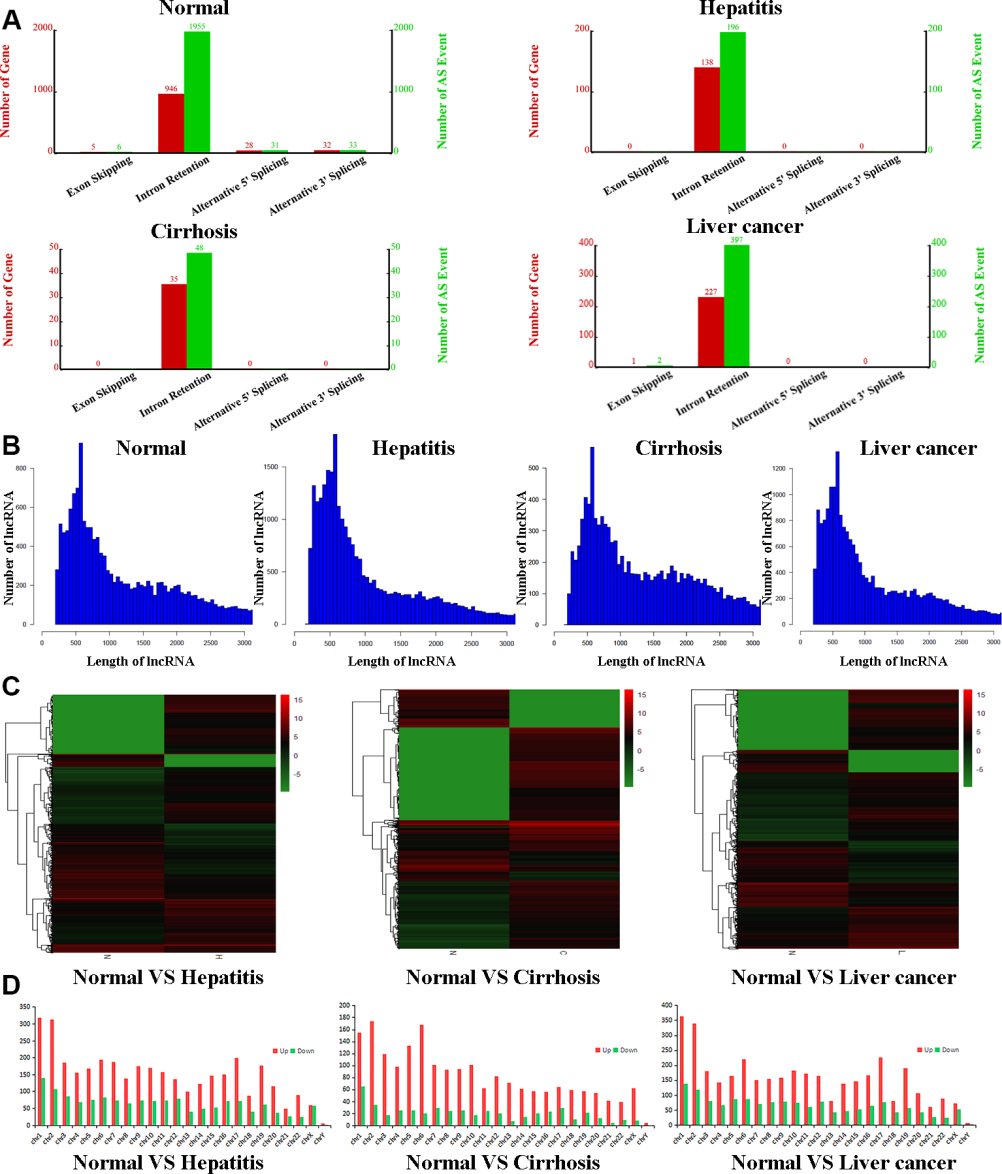
**Bioinformatic analyses of lncRNAs.** (**A**) Number of genes (red) and alternate splicing events (green) for lncRNAs from control, hepatitis, cirrhosis, and HCC groups. (**B**) Length distribution of lncRNAs in control, hepatitis, cirrhosis, and HCC groups. (**C**) Cluster heatmaps of lncRNA expression profiles in the control group compared to hepatitis, cirrhosis, and HCC groups. Gradient green represents downregulated lncRNAs, while gradient red represents upregulated lncRNAs. (**D**) Chromosomal distributions of lncRNAs in the control group compared to hepatitis, cirrhosis, and HCC groups. lncRNA, long noncoding RNA; HCC, hepatocellular carcinoma.

To further qualify differences between the exosomes of healthy volunteers and hepatic disease patients, we purified exosomal total RNA and conducted RNA-seq. RNAs were annotated, and lncRNAs were extracted from the data for further analysis. For all four groups, lncRNAs were mostly intronic. In the control group, we determined 946 relevant genes to be involved in lncRNA formation, with 1955 associated alternative splicing events (Figure 1A). In contrast, exosomes of the hepatitis, cirrhosis, and HCC groups had obviously fewer lncRNAs with fewer alternative splice events (ranging from 4- to 40-fold reduction), indicating that liver disease downregulates lncRNA production and alternative splicing of lncRNAs. However, exosomes of all four groups had similar length distributions for lncRNAs, the majority of which were ~500 nt (Figure 1B). The heatmap showed that compared to the control group, 34,175 lncRNAs were upregulated and 15,553 downregulated in the hepatitis group, 16,673 lncRNAs were upregulated and 22,033 downregulated in the cirrhosis group, and 28,475 lncRNAs were upregulated and 16,453 downregulated in HCC group (Figure 1C and Supplementary Tables 2–4). We also examined chromosomal distributions of lncRNAs. In the hepatitis, cirrhosis, and HCC groups, upregulated lncRNAs were most abundant on chromosomes 1 and 2, being about twice as much as downregulated lncRNAs. Interestingly, in the cirrhosis group, upregulated lncRNAs were also enriched on chromosomes 5 and 6 and were six times more than downregulated lncRNAs (Figure 1D), indicating that lncRNAs that play a role in liver disease are more distributed on chromosomes 1 and 2.

**Figure 2:**
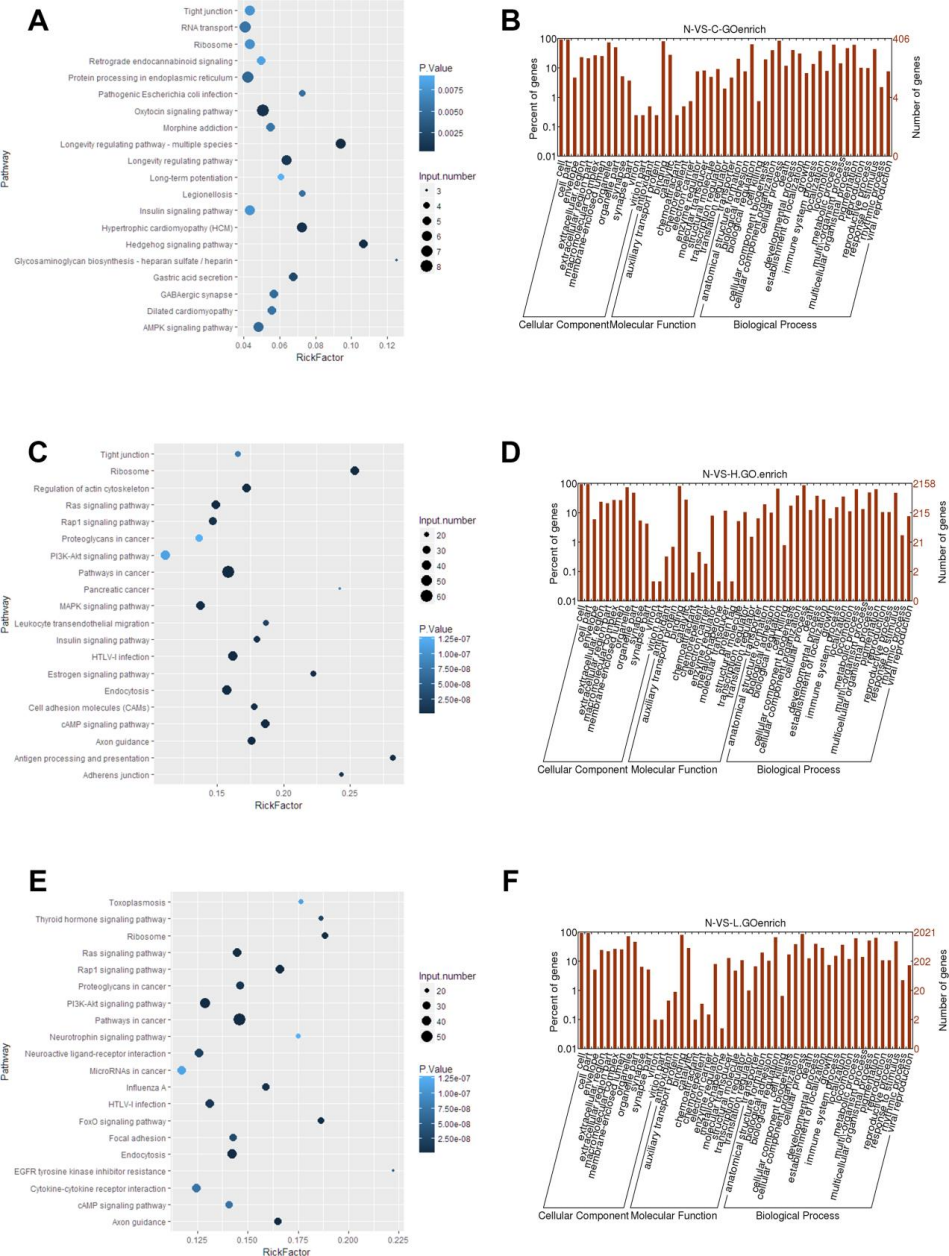
**Functional analyses of significantly expressed lncRNAs.** (**A**, **C**, **E**) KEGG pathway enrichment of the differentially expressed lncRNAs in control, hepatitis, and HCC groups. (**B**, **D**, **F**) GO analysis of differentially expressed lncRNAs in control, hepatitis, and HCC groups, respectively. lncRNA, long noncoding RNA; HCC, hepatocellular carcinoma.

**Figure 3:**
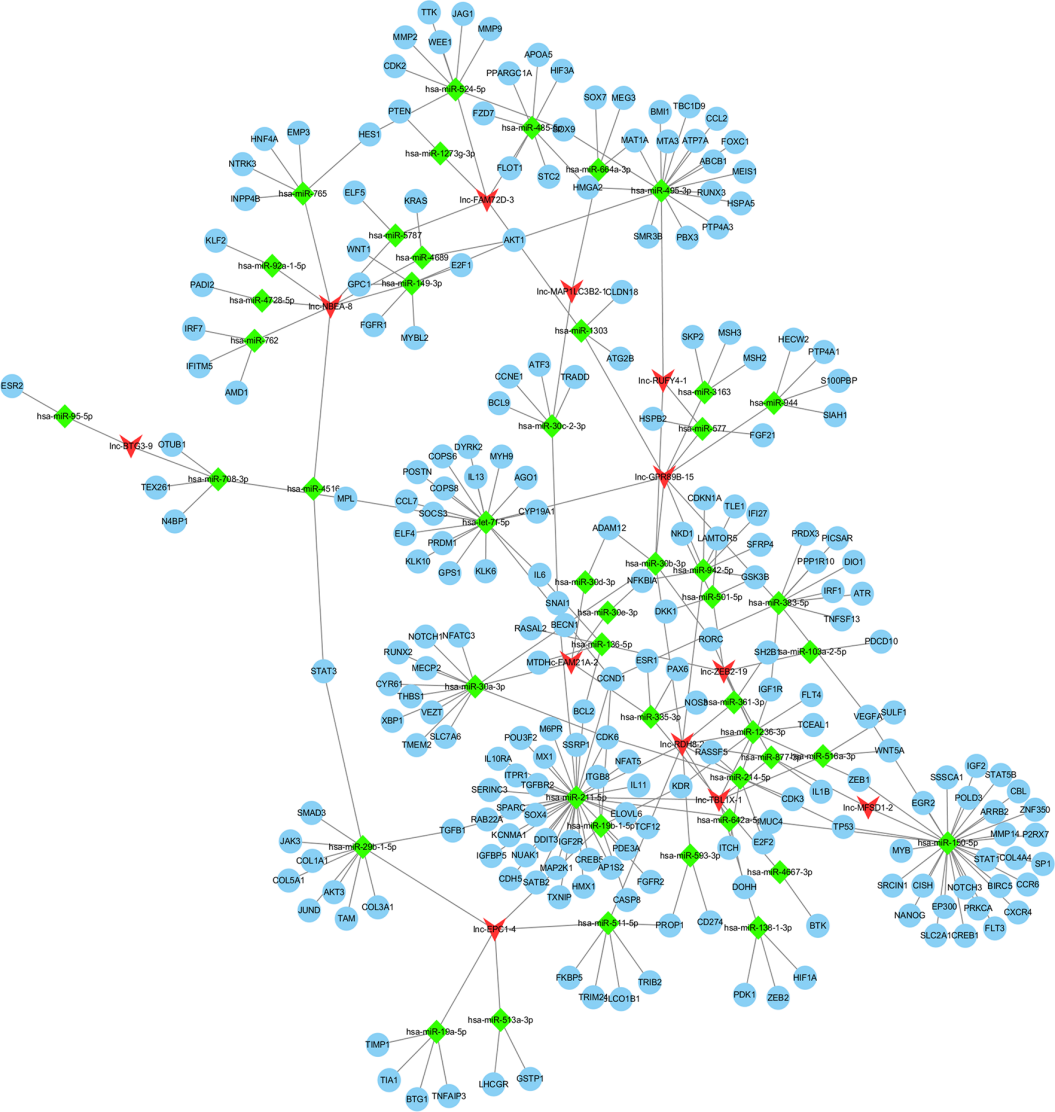
**Regulation network of differentially expressed lncRNAs. lncRNA, long noncoding RNA.**

To determine the potential relevance of differentially expressed lncRNAs from the hepatitis, cirrhosis, and HCC groups, we conducted KEGG and GO analysis. In KEGG pathway annotation, the pathways were mainly RNA transport and protein processing in the endoplasmic reticulum (Figure 2A): Ras, Rap1, and phosphatidylinositol-3 kinase/protein kinase B (PI3K/Akt) signaling pathways, which were enriched in the control group compared to hepatitis and HCC groups (Figure 2C, 2E). In addition, GO analysis showed that significantly expressed lncRNAs are more focused on the “cell” and “cell part” of cellular components, the “binding” of molecular function, and the “cellular process” and “biology regulation” of biological process in the control group compared to hepatitis and HCC groups (Figure 2B, 2D, 2F), indicating that pathways in KEGG analysis and biological processes in GO analysis play important roles in the occurrence and development of liver diseases. Regulatory networks of common significantly differentially expressed lncRNAs revealed many clusters with low interconnectedness between the genes (Figure 3), indicating that the underlying mechanism is complicated and that each lncRNA might perform distinct functions in regulating specific genes.

### Validation of the expression of differentially expressed lncRNAs

**Figure 4:**
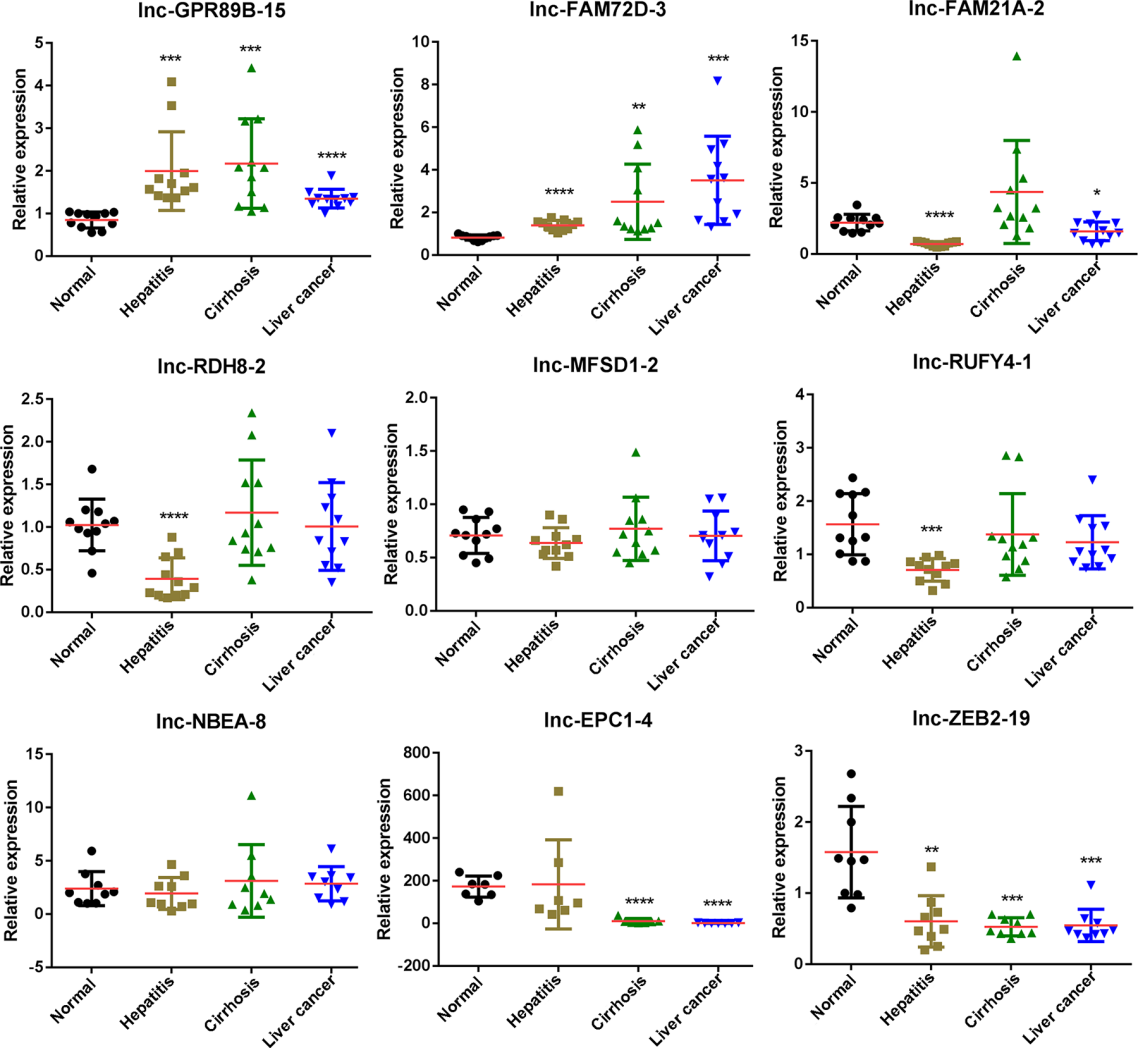
**Differentially expressed lncRNAs in exosomes of control, hepatitis, cirrhosis, and HCC groups validated by real-time qPCR.** lncRNA, long noncoding RNA; HCC, hepatocellular carcinoma; qPCR, quantitative polymerase chain reaction.

To further investigate the expression differences in exosomal lncRNAs of the four groups, we performed qPCR and examined the expression levels of nine lncRNAs. The expression levels of lnc-GPR89B-15 and lnc-ZEB2-19 verified by qPCR were consistent with RNA-seq results in hepatitis, cirrhosis, and HCC groups (Figure 4). lnc-FAM72D-3 expression in hepatitis, cirrhosis, and HCC groups was significantly high compared to the control group, while lnc-EPC1-4 expression in hepatitis, cirrhosis, and HCC groups was significantly low compared to the control group (Figure 4). Four lncRNAs (lnc-GPR89B-15, lnc-FAM72D-3, lncEPC1-4, and lnc-ZEB2-19) with differential expression in the HCC groups were selected for further evaluation.

**Figure 5:**
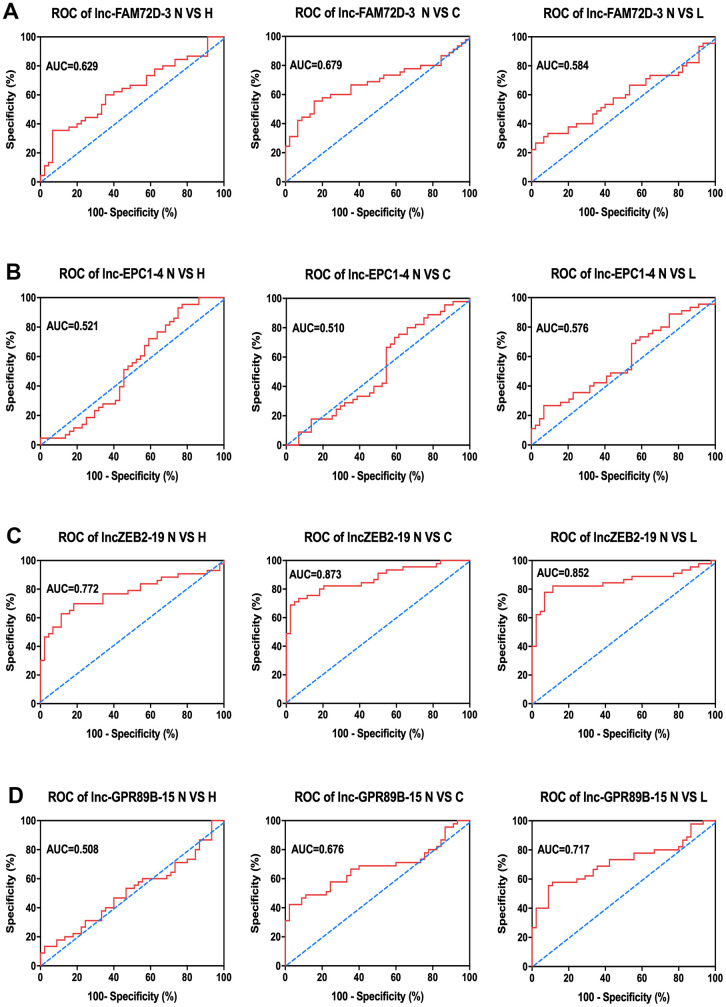
****Clinical analysis of AUC of (**A**) lnc-FAM72D-3, (**B**) lnc-EPC1-4, (**C**) lnc-ZEB2-19, and (**D**) lnc-GPR89B-15 in the control group compared to hepatitis, cirrhosis, and HCC groups. N VS H, normal versus hepatitis; N VS C, normal versus cirrhosis; N VS L, normal versus HCC; AUC, area under the curve; ROC, receiver operating characteristic; HCC, hepatocellular carcinoma.

Next, to determine the relationship between exosomal expression and diseases, we compared the expression levels of these lncRNAs between the control group and hepatitis, cirrhosis, and HCC groups (Table 2). As indicated by the area under the curve (AUC) of the receiver operating characteristic (ROC) curves, each of the four lncRNAs showed a close correlation with hepatic diseases Figure 5). Of them, lnc-ZEB2-19 had the highest AUC in all three comparisons, indicating a significant correlation between lnc-ZEB2-19 exosomal expression and hepatic diseases. Since most lncRNA expression levels were correlated with hepatitis, cirrhosis, and HCC to some extent, we combined the data from the patients and calculated the overall correlation between different patient characteristics and lncRNA expression (Table 3). The results suggested that the expression of lnc-EPC1-4 was significantly correlated with age, while the expression of lnc-GPR89B-15 and lnc-EPC1-4 was significantly correlated with AFP concentration (P < 0.05).

**Table 2:** Characteristics of the patients.

**Group**	**Variable**	**No.**	**Group**	**Variable**	**No.**
**Healthy (n=45)**	Age, years		Cirrhosis (n = 45)	Age, years	
	mean	29.608		mean	51.496
	SD	11.331		SD	10.339
	Male	26		Male	34
	female	19		female	11
	Serum AFP			Serum AFP	
	<20 ng/ml	45		<20 ng/ml	33
	≥20 ng/ml	0		=20–400 ng/ml	10
				>400 ng/ml	2
**Hepatitis (n=45)**	Age, years		liver cancer (n = 45)	Age, years	
	mean	41.143		mean	52.333
	SD	13.155		SD	11.909
	Male	38		Male	36
	female	7		female	9
	Serum AFP			Serum AFP	
	<20 ng/ml	20		<20 ng/ml	16
	=20–400 ng/ml	19		=20–400 ng/ml	12
	>400 ng/ml	6		>400 ng/ml	17

**Table 3:** Correlation between expression level of the differentially expressed lncRNAs and clinical characteristics.

**Variable**	**No. of case**	**lnc-FAM72D-3**			**lnc-EPC1-4**			**lnc-ZEB2-19**			**lnc-GPR89B-15**		
**Age (year)**		MEAN	R	P	MEAN	R	P	MEAN	R	P	MEAN	R	P
**<35**	57	2.847±0.185	0.468	0	21.842±3.410	0.165	0.02	1.68±0.217	−0.263	0	3.720±0.374	−0.266	0
**≥35**	123	12.075±1.076			42.430±5.82			0.77±0.057			2.638±0.148		
**Gender**													
**Male**	134	19.114±1.898	−0.493	0	40.992±6.441	−0.05	0.649	0.970±0.131	0.044	0.414	2.752±0.046	0.046	0.388
**Female**	46	4.099±0.307			63.036±0.20			1.108±0.101			32.801±5.274		
**AFP**													
**<20 ng/ml**	114		−0.069	0.197		0.16	0.003		−0.037	0.493		−0.118	0.027
**=20–400 ng/ml**	42												
**>400 ng/ml**	24												

Abbreviations: AFP, alpha fetoprotein; lnc-FAM72D-3, long non-coding RNA FAM72D-3; lnc-EPC1-4, long non-coding RNA; lnc-ZEB2-19, long non-coding RNA ZEB2-19; lnc-GPR89B-15 long non-coding RNA GPR89B-15.

### Function analysis of differentially expressed LncRNAs

**Figure 6:**
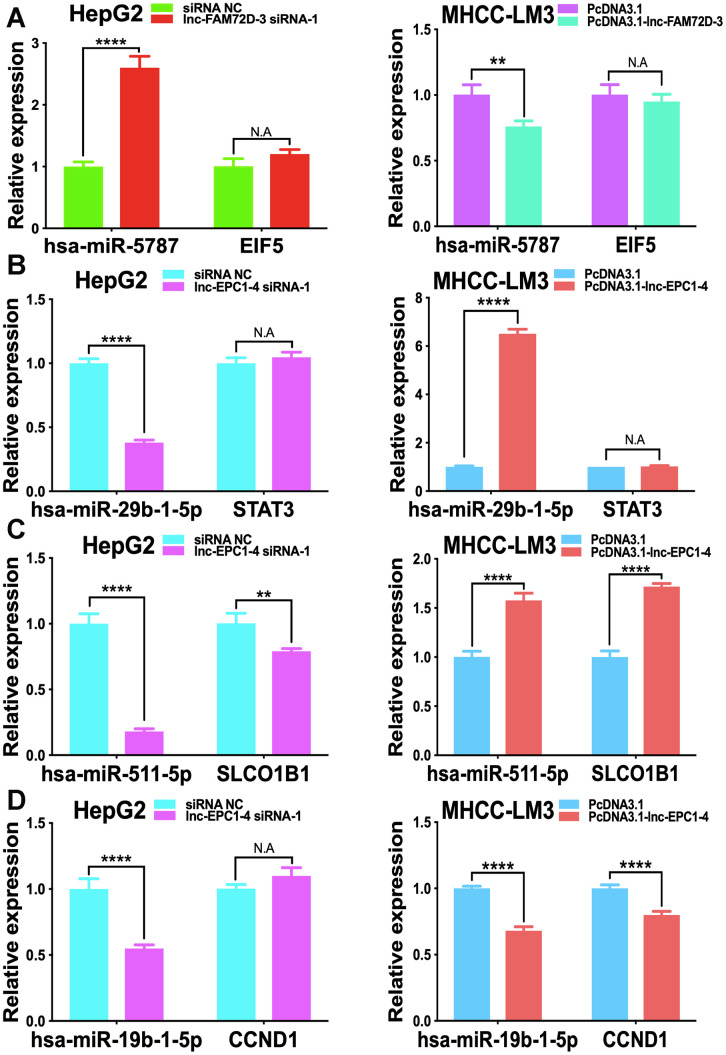
**Effect of lnc-FAM72D-3 and lnc-EPC1-4 suppression and overexpression on candidate targets.** (**A**) has-miR-5787 and EIF5 expression when lnc-FAM72D-3 was suppressed or overexpressed in HepG2 and MHCC-LM3 cells. (**B**) has-miR-29b-1-5p and STAT3 expression when lnc-EPC1-4 was suppressed or overexpressed in HepG2 and MHCC-LM3 cells. (**C**) has-miR-511-5p and SLCO1B1 expression when lnc-EPC1-4 was suppressed or overexpressed in HepG2 and MHCC-LM3 cells. (**D**) has-miR-19b-1-5p and CCND1 expression when lnc-EPC1-4 was suppressed or overexpressed in HepG2 and MHCC-LM3 cells. STAT3, signal transducer and activator of transcription 3.

**Figure 7:**
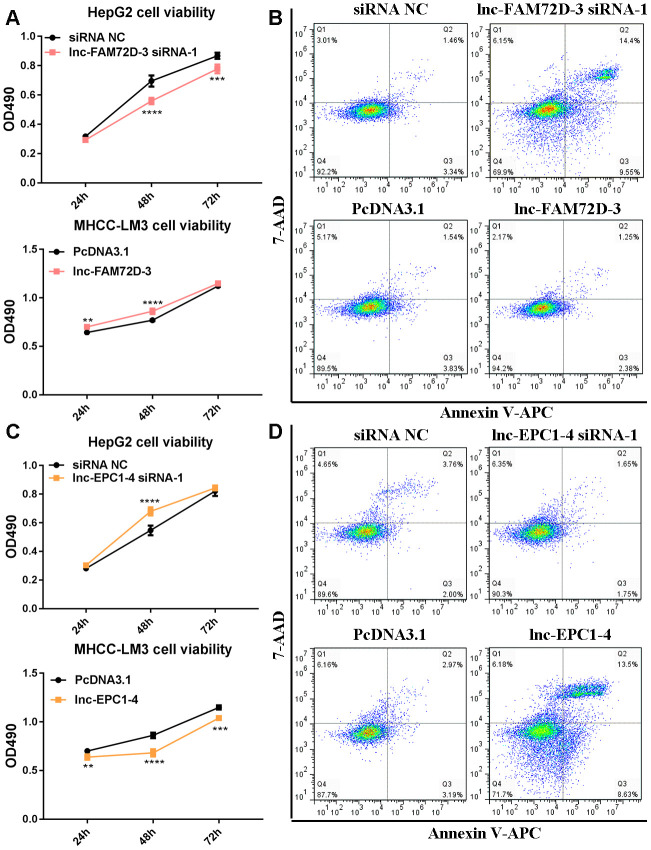
****(**A**) Cell viability and (**B**) cell apoptosis in cells with lnc-FAM72D-3 suppression or overexpression. (**C**) Cell viability and (**D**) cell apoptosis in cells with lnc-EPC1-4 suppression or overexpression.

The expression levels of lnc-GPR89B-15, lnc-FAM72D-3, lncEPC1-4, and lnc-ZEB2-19 were detected in different cell lines using qPCR (Supplementary Figure 2). lnc-FAM72D-3 and lnc-EPC1-4 expression was significantly different in different cell lines (Supplementary Figure 2). Therefore, we generated siRNAs and overexpression vectors to inhibit and promote lnc-FAM72D-3 and lnc-EPC1-4 expression. On the basis of their high expression levels, we selected HepG2 and SNU-423 cells for evaluation by siRNA-mediated knockdown, while on the basis of their low expression levels, we selected MHCC-LM3 and MHCC97-H cells for vector-mediated overexpression. The lnc-FAM72D-3 siRNA-1 significantly downregulated lnc-FAM72D-3 expression in HepG2 cells with a small but significant decrease in SNU-423 cells, while all three lnc-EPC1-4 siRNAs downregulated lnc-EPC1-4 expression (Supplementary Figure 3, left). Therefore, we selected lnc-FAM72D-3 siRNA-1 and lnc-EPC1-4 siRNA-1 for subsequent analysis (Supplementary Figure 3, right). qPCR results also confirmed that MHCC-LM3 and MHCC97-H overexpression was successful (>150-fold increase). Next, we identified candidate miRNAs predicted bioinformatically to be targeted by these lncRNAs. qPCR results confirmed that hsa-miR-5787 is targeted by lnc-FAM72D (Figure 6A). hsa-miR-29b-1-5p, hsa-miR-19b-1-5p, hsa-miR-511-5p, and SLCO1B1 levels were each significantly downregulated by lnc-EPC1-4 siRNA-1, while CCND1 levels were slightly upregulated (Figure 6B–6D). These results indicated that hsa-miR-5787 might be a target of lnc-FAM72D-3, while lnc-EPC1-4 might target several genes, including *hsa-miR-29b-1-5p*, *hsa-miR-511-5p*, and *SLCO1B1*. To further assess the functional effects of lnc-FAM72D-3 expression modulation, we performed cell proliferation and apoptosis assays. HepG2 cells transfected with lnc-FAM72D-3 siRNA-1 or lnc-EPC1-4 siRNA-1 and MHCC-LM3 cells transfected with pcDNA3.1-FAM72D-3 or pcDNA3.1-EPC1-4 were evaluated by MTS and flow cytometry assays. When HepG2 cells were transfected with lnc-FAM72D-3 siRNA-1 for 48 h, ~50% of HepG2 cells were viable, which was low compared to the siRNA negative control (NC) group, indicating that lnc-FAM72D-3 siRNA-1 significantly inhibits HepG2 cells proliferation (Figure 7A). In addition, pcDNA3.1-FAM72D-3 enhanced HepG2 cell viability. Also, the early apoptotic percentage of HepG2 cells transfected with lnc-FAM72D-3 siRNA-1 was 9.55%, which was low compared to the siRNA NC group, indicating that lnc-FAM72D-3 siRNA-1 induces HepG2 cell apoptosis. In addition, pcDNA3.1-FAM72D-3 induced slight inhibition of cell apoptosis compared to the pcDNA3.1 group (Figure 7B). Lnc-EPC1-4 siRNA and pcDNA3.1-EPC1-4 had the opposite effect: lncEPC1-4 siRNA increased cell viability and decreased apoptosis, while pcDNA3.1-EPC1-4 decreased cell viability and increased apoptosis (Figure 7C, 7D). These results indicated that *lnc-FAM72D-3* functions as an oncogene that is upregulated in HCC, while *lnc-EPC1-4* functions as a tumor suppressor gene that is downregulated in HCC.

## DISCUSSION

In this study, we examined exosomal lncRNAs that are differentially expressed in hepatic disease patients. Increased lnc-FAM72D-3 and decreased lnc-EPC1-4 levels are clearly correlated with HCC; these findings were verified by qPCR for a group of 180 healthy volunteers and hepatic disease patients. Functional validation with MTS and cell apoptosis assays showed that lnc-FAM72D-3 knockdown induces significant inhibition of cell proliferation and promotes cell apoptosis, while upregulation of lnc-EPC1-4 expression has the same effect. There are currently no reports on the exact underlying mechanism; however, we have elucidated new roles for them in HCC and other hepatic diseases. On the basis of our bioinformatic prediction of downstream targets of lnc-FAM72D-3 and lnc-EPC1-4, several candidate target genes and miRNAs have been shown to regulate cell proliferation and cell apoptosis. These included hsa-miR-511-5p, which we had demonstrated increases by pcDNA3.1-lnc-EPC1-4 transfection. Consistent with our findings, *miR-511* acts as a tumor suppressor gene that attenuates tumor cell growth and proliferation by inhibiting tribbles pseudokinase 2 (TRIB2) [25]. TRIB2 is involved in the etiology of various cancers, including leukemia, melanoma, lung cancer, and HCC. In addition, miR-511 regulates the AKT signaling module, which plays a pivotal function in cell proliferation, differentiation, metabolism, and apoptosis in HCC [26–29]. MiR-29b, which we had shown is induced by pcDNA3.1-lnc-EPC1-4 and inhibited by lnc-EPC1-4 siRNA, has also been suggested to participate in the inhibition of angiogenesis and tumorigenesis of cancer through the Akt3 signaling pathway [30]. Therefore, decreased lnc-EPC1-4 siRNA levels in HCC might enhance cell growth by activating the AKT pathway.

We also demonstrated that SLCO1B1 is targeted by lnc-EPC1-4. The sequential administration of sorafenib (front-line treatment) and regorafenib (second-line treatment) efficiently improves therapeutic efficacy in advanced HCC. Influx transporters (SLCO1B1, SLCO1B3, and SLC22A1) are highly involved in the metabolism and clearance of these chemotherapy agents in the liver, while downregulation of these influx transporters might inhibit cellular uptake, thereby discounting their clinical benefits [31–33]. Hu et al. [31] reported that SLCO1B1 expression is significantly downregulated in HCC, which is consistent with our findings. In addition, *CDCA7*, as a target gene of hsa-miR-29b-1-5p, is overexpressed in several malignancies [34, 35]. lnc-EPC1-4 overexpression induces miR-29b-1-5p expression, which provides a potential mechanism for CDCA7 overexpression and might also explain, in part, the antitumor effects of lnc-EPC1-4.

Our results also demonstrated that lnc-FAM72D-3 siRNA-1 induces hsa-miR-5787 expression, while pcDNA3.1-lnc-FAM72D-3 inhibits hsa-miR-5787 expression. However, modulation of lnc-FAM72D-3 fails to affect eukaryotic translation initiation factor 5 (EIF5), which is a target of has-miR-5787 [36]. EIF5 not only plays an important role in the initiation of translation of some proteins but also regulates the occurrence of cancer and promotes cell proliferation, senescence, and apoptosis. Therefore, a mechanism that involves alternate hsa-miR-5787 targets might account for the oncogenic effects of lnc-FAM72D-3 in HCC cells.

In summary, lnc-FAM72D-3 and lnc-EPC1-4 might be used as diagnostic and prognostic biomarkers for HCC. Future studies that characterize the expression of additional candidate targets of these lncRNAs might help us better define their mechanisms, which might ultimately lead to the development of new diagnostic and therapeutic tools for HCC.

## MATERIALS AND METHODS

### Exosome purification from serum

Whole blood was collected from 45 healthy volunteers (controls), 45 hepatitis patients, 45 cirrhosis patients, and 45 HCC patients. Serum was isolated from the whole blood and ultracentrifuged using a Beckman L8 ultracentrifuge (GMI, Ramsey, MN, USA) at 100,000×*g* for 20 h at 10°C. The supernatants were discarded, and pellets containing exosomes were resuspended in phosphate buffer solution (PBS). The size of the exosomes was validated by using nanoparticle tracking analysis (NTA; Particle Metrix, Germany), and the content of membrane proteins, such as CD63, CD9, and TSG101, was verified using western blotting.

### Western blotting

Exosomes, HepG2 cells, and MHCC-LM3 cells were lysed in 1% sodium dodecyl sulfate (SDS) lysis buffer. Protein concentrations were evaluated using the bicinchoninic acid (BCA) protein assay kit (Beyotime, Shanghai, China). Proteins were subjected to 10% sodium dodecyl sulfate–polyacrylamide gel electrophoresis (SDS-PAGE) and then transferred onto nitrocellulose membranes. The nitrocellulose membranes were blocked with 5% nonfat milk in PBS for 1 h at room temperature and incubated overnight at 4°C with primary antibodies anti-CD63 (ab134045; Abcam, UK), anti-TSG101 (ab125011; Abcam), anti-CD9 (ab92726; Abcam), and anti-calnexin (ab22595; Abcam). After several washes, the nitrocellulose membranes were incubated in a blocking buffer with a secondary antibody coupled to horseradish peroxidase (HRP) for 2 h at room temperature. The complexes were detected by electrochemiluminescence (ECL) (Amersham Biosciences/GE Healthcare, Velizy, France).

### Cell culture

HCC cell lines (HepG2, Hep3B, SNU-423, and PLC/PRF/5) were obtained from the American Type Culture Collection (ATCC, Manassas, VA, USA). The human normal liver L02 cell line and Huh-7, MHCC-LM3, and MHCC97-H cells were obtained from CELLCOOK (Guangzhou, China) and cultured with 5% CO_2_ at 37°C. The cells were maintained in high-glucose Dulbecco’s Modified Eagle Medium supplemented with 10% fetal bovine serum (FBS; Gibco, Thermo Fisher Scientific, Waltham, MA, USA) and passaged every 3 days.

### LncRNA suppression and overexpression

Full-length lncRNAs were amplified by polymerase chain reaction (PCR) using Thermo Scientific Phusion Flash High-Fidelity PCR Master Mix (Thermo Fisher Scientific), and corresponding complementary DNAs (cDNAs) were subcloned into the NheI and KpnI sites of pcDNA3.1(+) (Genepharma, China) according to the manufacturer’s instructions. The vectors constructed were verified by Sanger sequencing. Small interfering RNAs (siRNAs) for candidate lncRNAs were designed and synthesized. The vectors and siRNAs were transfected into cell lines using lipofectamine 2000 (Thermo Fisher Scientific) according to the manufacturer’s instructions. The expression level of lncRNAs and their targeted miRNAs and messenger RNAs (mRNAs) were determined by real-time quantitative PCR (qPCR) (Supplementary Table 1).

### MTS assay

Cells were seeded at a density of 1 × 10^6^/mL into 96-well plates (100 μL/well), treated with overexpression vectors or siRNAs, and examined after 24, 48, and 72 h. A 3-(4,5-dimethylthiazol-2-yl)-5-(3-carboxymethoxyphenyl)-2-(4-sulfophenyl)-2H-tetrazolium (MTS) mixture (Promega Corporation, Madison, WI, USA) was added for 3 h, and the optical density of the cells was detected at a frequency of 490 nm.

### Cell apoptosis assay

Cells were resuspended in 1× binding buffer, and 5 μL of fluorochrome-conjugated annexin V and 5 μL of 7-aminoactinomycin D (7-AAD) staining solution (Thermo Fisher Scientific) were added. The cells were assessed using a fluorescence-activated cell sorting Calibur flow cytometer, and the percentage of the apoptotic cells was measured.

### RNA-seq

Total RNA was purified from exosomes from the isolated serum from 45 healthy volunteers (controls), 45 hepatitis patients, 45 cirrhosis patients, and 45 HCC patients using the RNeasy Mini Kit (QIAGEN, Germany). RNA integrity was evaluated on the basis of the RNA integrity number (RIN) using Agilent Bioanalyzer 2100 (Agilent Technologies, Santa Clara, CA, USA). RNA was further purified using the RNA Clean XP Kit (Beckman Coulter, Brea, CA, UA), and the DNA residue was removed using the RNase-free DNase Set (QIAGEN). RNA quality and concentration were determined using NanoDrop 2000 (Thermo Fisher Scientific). Ribosomal RNA (rRNA) was removed using the NEBNext rRNA Depletion kit (New England Biolabs, Ipswich, MA, USA). Then, 1 μg of total RNA was used for library preparation using the VAHTSTM mRNA-seq v2 library Prep Kit (Vazyme, Nanjing, China) according to the manufacturer’s instructions. RNA was fragmented and then double-stranded cDNA was synthesized. End-polishing was performed, and the cDNA fragments were ligated with adapters and then subjected to universal PCR amplification in order to obtain a sufficient library for sequencing. The library quality was assessed using Agilent Bioanalyzer 2100. RNA-seq was performed using Illumina Hiseq 4000 (Illumina, San Diego, CA, USA). The data were assembled and annotated with corresponding symbols for transcripts. Differentially expressed lncRNAs were screened using R software (*q* < 0.05 and fold change > 2.0).

### Gene ontology (GO) and Kyoto encyclopedia of genes and genomes (KEGG) pathway analysis

The Database for Annotation, Visualization and Integrated Discovery was used to annotate the potential functions of various signaling pathways for differentially expressed lncRNAs. The functional annotations of parental genes were predicted by GO functional annotation. Scatter plots were used to visualize GO analysis results. KEGG pathway annotation was used to identify relevant pathways of differentially expressed lncRNAs.

### Construction of an lncRNA-miRNA-mRNA network

We predicted miRNA binding to lncRNA using miRanda software. By inputting miRNA and lncRNA sequences, we predicted the miRNA that lncRNA might combine with and filtered out the miRNA that was differentially expressed in RNA-seq. miRNA target genes were downloaded in miRTarbase, while an lncRNA-miRNA-mRNA regulatory network was constructed using Cytoscape ver. 3.5.1.

### qPCR

Total RNA was extracted from exosomes and gene expression verified by PCR. cDNA was synthesized from total RNA using M-MLV Reverse Transcriptase (Promega Corporation) according to the manufacturer’s instructions. PCR was performed using the GoTaq qPCR Master Mix (Promega Corporation). PCR amplification was performed using the ABI 7500 system (Applied Biosystems, Foster City, CA, USA). Glyceraldehyde 3-phosphate dehydrogenase (GAPDH) was used as an internal control.

### Statistical analysis

Statistical analysis was performed using SPSS Statistics 19 (IBM Corporation. Armonk, NY, USA) and GraphPad Prism (GraphPad Software, CA, USA). Comparisons were performed using independent *t*-tests, one-way analysis of variance (ANOVA), and two-way ANOVA when data corresponded to a normal distribution. *P* < 0.05 was considered statistically significant.

### Ethics statement

This study was approved by the ethics committee of Third Affiliated Hospital of Sun Yat-sen University. The patients provided informed written consent.

## Supplementary Material

Supplementary Figures

Supplementary Table 1

Supplementary Table 2

Supplementary Table 3

Supplementary Table 4
