# Supplementary material for: Serum exosomal long noncoding RNAs lnc-FAM72D-3 and lnc-EPC1-4 as diagnostic biomarkers for hepatocellular carcinoma
Source: Aging (Albany NY). 2020 Jun 18;12(12):11843–63. doi: 10.18632/aging.103355 (PMC7343450; doi:10.18632/aging.103355)
Supplement: Supplementary Figures [file aging-12-103355-s002..pdf]

# SUPPLEMENTARY FIGURES

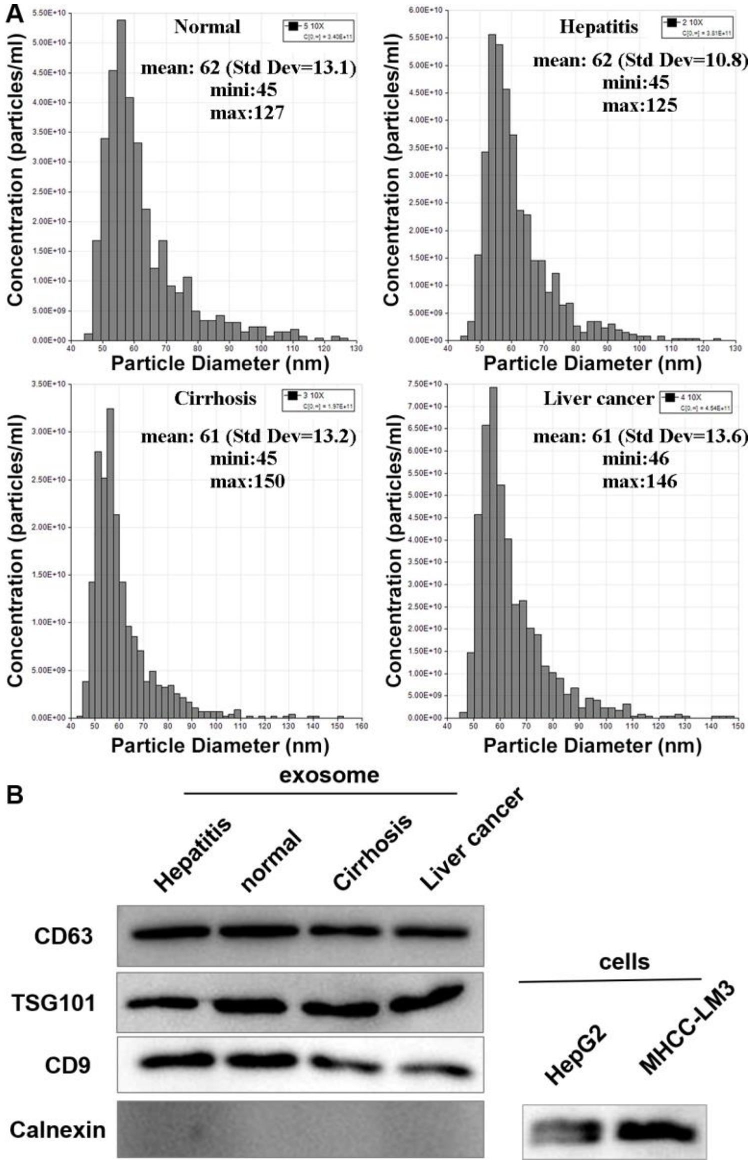


**Supplementary Figure 1. Validation of exosome purification.** (**A**) Size distribution of exosomes in control, hepatitis, cirrhosis, and HCC groups. (**B**) Western blot analysis of exosome-specific proteins (CD63, TSG101, and CD9) in control, hepatitis, cirrhosis, and HCC groups and calnexin protein analysis in control, hepatitis, cirrhosis, and HCC groups and HepG2 and MHCC-LM3 cells. HCC, hepatocellular carcinoma.

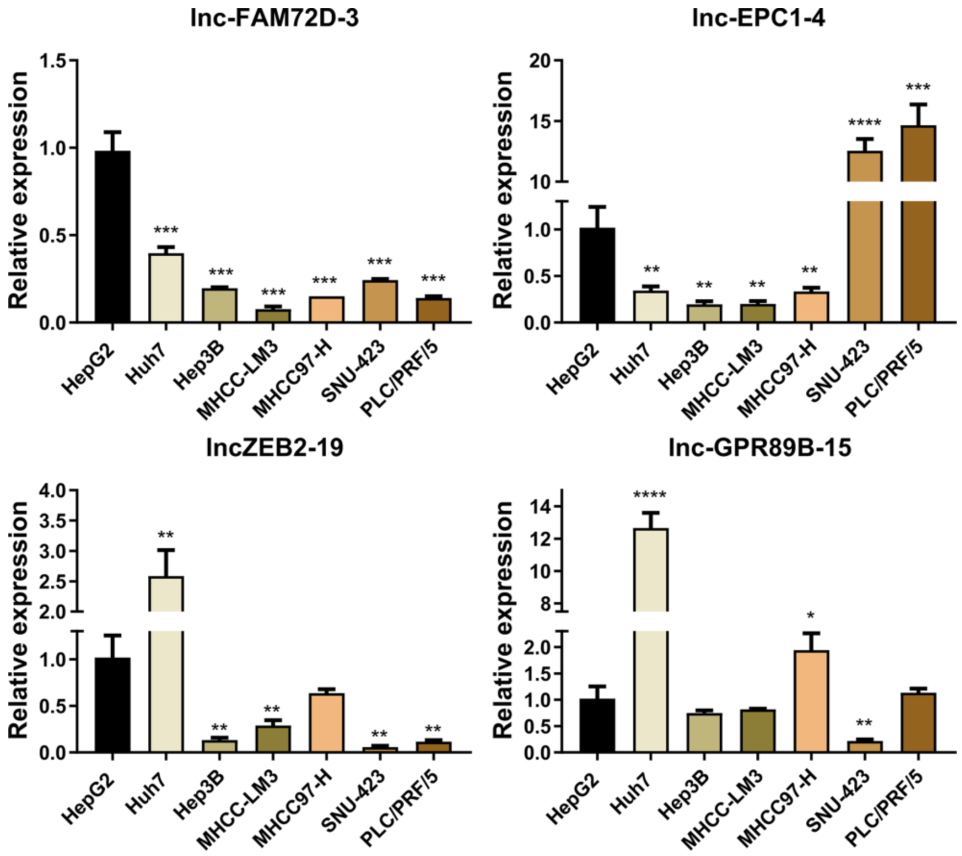


**Supplementary Figure 2. Relative lnc-FAM72D-3, lnc-EPC1-4, lnc-ZEB2-19, and lnc-GPR89B-15 expression in different cell lines determined by real-time qPCR. qPCR, quantitative polymerase chain reaction.**

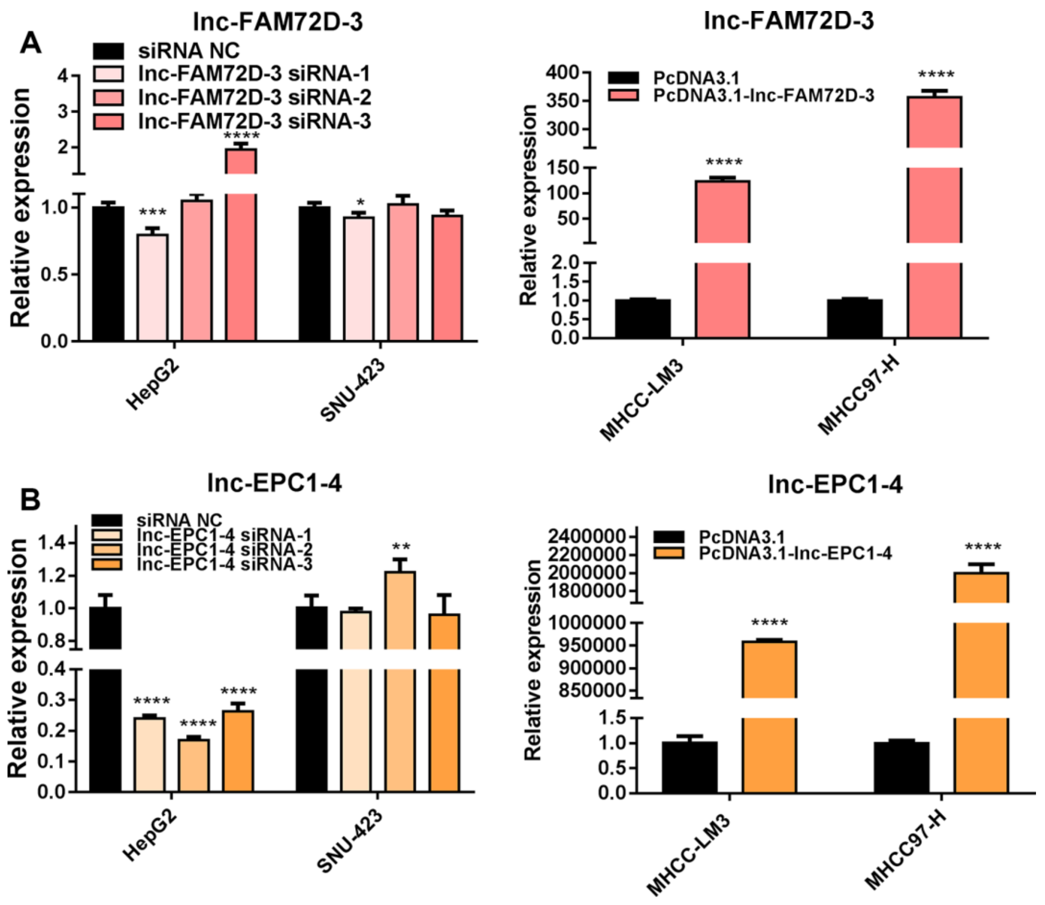


**Supplementary Figure 3. Suppression and overexpression of lnc-FAM72D-3 and lnc-EPC1-4 in different cells.** (**A**) lnc-FAM72D-3 expression in different cells treated with siRNA and lnc-FAM72D-3 overexpression vector. (**B**) lnc-EPC1-4 expression in different cells treated with siRNA and lnc-EPC1-4 overexpression vector. siRNA, small interfering RNA.
