# Supplementary material for: Serum exosomal long noncoding RNAs lnc-FAM72D-3 and lnc-EPC1-4 as diagnostic biomarkers for hepatocellular carcinoma
Source: Aging (Albany NY). 2020 Jun 18;12(12):11843–63. doi: 10.18632/aging.103355 (PMC7343450; doi:10.18632/aging.103355)
Supplement: Supplementary Table 1 [file aging-12-103355-s001..pdf]

SUPPLEMENTARY TABLE

Supplementary Table 1. LncRNA and miRNA primer sequence for quantitative real time (qPCR).

lncRNA	Noncode transcript ID	Primer sequence
lnc-RUFY4-1	NONHSAT076871.2-F	ACAGGTTAAGGGAACGCGAA
	NONHSAT076871.2-R	CAGAAATCCACCAGCCAGCA
lnc-MFSD1-2	NONHSAT092952.2-F	AATGGATTGGGGGTTGAGGG
	NONHSAT092952.2-R	CCGACCTCACATCCAGCAAA
lnc-RDH8-2	NONHSAT061016.2-F1	CCTGCAAACTGGAACTGGGA
	NONHSAT061016.2-R1	GCCGGTAAGAAGGAGATGGAG
lnc-GPR89B-15:1	Inc-GPR8913-151-F	GGGTGCTAAGGTCGCAATGA
	Inc-GPR8913-151-R	GCATAATTCAATGGGCAGCAGT
lnc-FAM72D-3	NONHSAT149711.1-F	TGCCAGGGTTGGACCTAAGA
	NONHSAT149711.1-R	AGTAGGAGTGGGGTACTGGC
lnc-NBEA-8	NONHSAT165530.1-F2	TGAGGTCTCTTGTTCCCTACC
	NONHSAT165530.1-R2	CCAGGAAAGATGTCAAAGGAAAA
lnc-ZEB2-19	NONHSAT074841.2-F	GCGACCTTTGGGAGCTACA
	NONHSAT074841.2-R	CCTCAGTTGTAGAGGCAGCTT
lnc-EPC1-4	NONHSAT156862.1-F1	AAGTGTGGCCAGAGAAGAAGAC
	NONHSAT156862.1-R1	TCAGTGGCAGTTGTTTCAGGTT
lnc-FAM21A-2	NONHSAT155490.1-F	ATTTGGCATGTGCTTGGGATAC
	NONHSAT155490.1-R	GCAGTGAGAGAAACCTATGCCT
**miRNA**		**Primer sequence**
hsa-miR-5787-F		TTAGGGCTGGGGCGCGG
hsa-miR-5787-RT		GTCGTATCCAGTGCAGGGTCCGAGGTATTCGCACTGGATACGACACCTCC
hsa-miR-29b-1-5p-F		GCTGGTTTCATATGGTGG
hsa-miR-29b-1-5p-RT		GTCGTATCCAGTGCAGGGTCCGAGGTATTCGCACTGGATACGACTCTAAA
hsa-miR-19b-1-5p-F		GCGAGTTTTGCAGGTTTGCA
hsa-miR-19b-1-5p-RT		GTCGTATCCAGTGCAGGGTCCGAGGTATTCGCACTGGATACGACGCTGGA
hsa-miR-511-5p-RT		GCGGTGTCTTTTGCTCTG
hsa-miR-511-5p-F		GTCGTATCCAGTGCAGGGTCCGAGGTATTCGCACTGGATACGACTGACTG
H-SLCO1B1-F		ACTGATTCTCGATGGGTTGG
H-SLCO1B1-R		TTTCCAGCACATGCAAAGAC
H-ELF5-F		GTTCTGCTGCGACCAGTACA
H-ELF5-R		TGCCACTTGTTTTCAAGCAG
H-STAT3-F		ACCTGCAGCAATACCATTGAC
H-STAT3-R		AAGGTGAGGGACTCAAACTGC
H-CCND1-F		TCCTCTCCAAAATGCCAGAG
H-CCND1-R		GGCGGATTGGAAATGAACTT
lnc-FAM72D-3 siRNA-1		GAGAATGAGTAGAGCCCTT
lnc-FAM72D-3 siRNA-2		GGGACTGGGATGAAGATTT
lnc-FAM72D-3 siRNA-3		GCAGTATCCATATATGTCA
lnc-EPC1-4 siRNA-1		GCACTAATGGGAGCTCTGT
lnc-EPC1-4 siRNA-2		GAAGCAACATCAAGATGAA
lnc-EPC1-4 siRNA-3		GAGAAAGACAGTAGGTCAA
